# Chemical Analysis and Biological Activity of the Essential Oils of Two Valerianaceous Species from China: *Nardostachys chinensis* and *Valeriana officinalis*

**DOI:** 10.3390/molecules15096411

**Published:** 2010-09-14

**Authors:** Jihua Wang, Jianglin Zhao, Hao Liu, Ligang Zhou, Zhilong Liu, Jingguo Wang, Jianguo Han, Zhu Yu, Fuyu Yang

**Affiliations:** 1 College of Agronomy and Biotechnology, China Agricultural University, Beijing 100193, China; 2 College of Resources and Environmental Sciences, China Agricultural University, Beijing 100193, China; 3 College of Animal Science and Technology, China Agricultural University, Beijing 100193, China

**Keywords:** Valerianaceae, *Nardostachys chinensis*, *Valeriana officinalis*, essential oil, antimicrobial activity, antioxidant activity

## Abstract

In order to investigate essential oils with biological activity from local wild plants, two valerianaceous species, *Nardostachys chinensis* and *Valeriana officinalis*, were screened for their antimicrobial and antioxidant activity. The essential oils were obtained from the roots and rhizomes of the two plants by hydro-distillation, and were analyzed for their chemical composition by gas chromatography (GC) and gas chromatography-mass spectrometry (GC-MS). Calarene (25.31%), aristolone (13.35%), α-selinene (7.32%) and β-maaliene (6.70%) were the major compounds of the 23 identified components which accounted for 92.76% of the total oil of *N. chinensis*. Patchoulol (16.75%), α-pinene (14.81%), and β-humulene (8.19%) were the major compounds among the 20 identified components, which accounted for 88.11% of the total oil of *V. officinalis*. Both oils were rich in sesquiterpene hydrocarbons as well as their oxygenated derivatives. Essential oils were shown to have broad spectrum antibacterial activity with MIC values that ranged from 62.5 μg/mL to 400 μg/mL, and IC_50_ values from 36.93 μg/mL to 374.72 μg/mL. The oils were also shown to have moderate antifungal activity to *Candida albicans* growth as well as inhibition of spore germination of *Magnaporthe oryzae*. Two essential oils were assessed by 1,1-diphenyl-2-picrylhydrazyl (DPPH) free radical scavenging, β-carotene bleaching and ferrozine-ferrous ions assays, respectively, to show moderate antioxidant activity. Results suggest that the isolated essential oils could be used for future development of antimicrobial and antioxidant agents.

## 1. Introduction

Essential oils are complex mixtures of natural compounds comprised mostly of volatile constituents like lipids, terpenoids, ketones, phenols and oxygenated derivatives with multiple biological activities such as antimicrobial, insecticidal and antioxidant properties [1,2,3]. Essential oils have received much attention in the prevention of plant and animal diseases as well as for preventing oxidative damage by reactive oxygen and nitrogen species (ROS/RNS) [4,5,6,7]. In recent years, increasing attention has been paid to the exploration of naturally-occurring antioxidants and antimicrobials because of the growing consumer demand for food products free from synthetic chemical additives. The plant kingdom has attracted special interest because of its remarkable diversity in producing natural compounds.

*Nardostachys chinensis* Batal. and *Valeriana officinalis* L. are two valerianaceous species [8]. *N. chinensis* is a perennial herb that grows in West and Northwest China. In traditional Chinese medicine, the roots and rhizomes of *N. chinensis* are used for their stomachic and sedative effects [9]. The plant has been known to be rich in sesquiterpenoids, which were found to exhibit antimalarial, antinociceptive, and cytotoxic activities [10], as well as to be the enhancer of neurite outgrowth-promoting activity [11]. *V. officinalis* is also a perennial herb which roots and rhizomes have been used in traditional Chinese medicine for their sedative and antispasmodic properties [9]. The major groups of constituents in this plant are valepotriates and sesquiterpenes. It is not yet clearly understood which components of *V. officinalis* are responsible for the therapeutic properties [12].

To the best of our knowledge, antimicrobial and antioxidant activities of the essential oils of *N. chinensis* and *V. officinalis* from China have not been reported, although there were some papers that clarified the components of these essential oils including monoterpenes, sesquiterpenes, and their oxygenated derivatives [13,14,15,16,17,18,19,20]. The aim of the present study was to analyze the chemical composition of the essential oils of *N. chinensis* and *V. officinalis* from China as well as to evaluate their antioxidant activity and *in vitro* antimicrobial activity against selected plant pathogens.

## 2. Results and Discussion

### 2.1. Essential oil analysis

After extraction, the yields (v/w) of the essential oils of *N. chinensis* and *V. officinalis* were calculated as 0.9% and 0.7%, respectively. Oils were analyzed for chemical composition by GC and GC-MS. For *N. chinensis*, at least 23 compounds were identified, which accounted for 92.76% of the total oil (Table 1). The major compounds in the oil were calarene (25.31%), aristolone (13.35%), α-selinene (7.32%), β-maaliene (6.70%), and spathulenol (6.28%). Both sesquiterpene hydrocarbons and oxygenated sesquiterpenes constituted the dominant portions which accounted for 53.17% and 34.24%, respectively. The chemical profiles of the essential oil of *N. chinensis* in this study were similar to those of the previous report [*i.e*. the main compound in the oil was calarene (37.7%)] [19].

Twenty compounds were identified which accounted for 88.11% of the total oil of *V. officinalis* (Table 1). The most abundant compounds were patchoulol (16.75%), α-pinene (14.81%), β-humulene (8.19%) and α-bulnesene (7.10%). Both monoterpene and sesquiterpene hydrocarbons constituted the dominant portions, which accounted for 27.88% and 34.10%, respectively. The chemical profiles of the *V. officinalis* essential oil in this study was different from those of previous reports [13,14,17] (*i.e*., the content of bornyl acetate was analyzed as 11.3% by Pavlovic *et al*. [13], and the patchoulol content was only 1.2% in the oil analyzed by Huang *et al*. [17]). The differences may be attributed to a different geographical environment, cultivar type, seasonality, physiological age of the plant, and the method of oil isolation [12]. α-Bulnesene has also been found in *Stachys sericea* [26] and *Eucalyptus* species [27].

**Table 1:** Chemical composition of the essential oils from *N. chinensis* and *V. officinalis*.

Compound^a^	RI^b^	RA (%)^c^ in *N. chinensis*	RA (%)^c^ in *V.* *officinalis*
α-Pinene	927	t	14.81
Camphene	941	-	6.51
*p*-Cymene	1010	0.11	-
D-Limonene	1014	-	6.56
Eucalyptol	1017	0.52	-
γ-Terpinene	1047	t	-
Terpinene-4-ol	1175	t	-
*O*-Methylthymol	1235	t	-
*O*-Methylcarvacrol	1245	0.15	-
Bornyl acetate	1289	-	6.73
1,2,3-Trimethylindene	1324	0.14	-
β-Patchoulene	1388	0.63	2.32
β-Elemene	1396	0.24	0.83
10-Epicyperene	1407	0.24	-
β-Maaliene	1418	6.70	-
β-Caryophyllene	1426	-	1.17
Aristolene	1427	3.74	-
*trans*-α-Farnesene	1440	-	0.34
α-Guaiene	1442	-	3.62
Calarene	1444	25.31	-
Epizonarene	1450	0.90	-
α-Caryophyllene	1461	-	1.90
β-Humulene	1464	-	8.19
α-Gurjunene	1465	5.72	-
α-Elemene	1468	-	1.21
Selina-4,11-diene	1481	-	0.42
*trans*-β-Ionone	1491	3.81	-
β-Helmiscapene	1493	-	1.11
δ-Selinene	1501	-	2.68
α-Bulnesene	1501	1.38	7.10
Bornyl isovalerate	1521	-	1.31
α-Panasinsene	1526	0.47	3.21
δ-Amorphene	1529	0.52	-
1,2-Diisopropylbenzene	1572	0.62	-
α-Selinene	1577	7.32	-
Spathulenol	1587	6.28	-
Globulol	1593	2.96	-
1,2-Dimethyl-4-formyl-1-cyclohexene	1617	-	1.34
Patchoulol	1672	5.87	16.75
Valeranone	1685	5.78	-
Aristolone	1776	13.35	-
Total identified		92.76	88.11
Monoterpene hydrocarbons		0.11	27.88
Oxygenated monoterpenes		0.67	9.38
Sesquiterpene hydrocarbons		53.17	34.10
Oxygenated sesquiterpenes		34.24	18.14

t: trace (0.05-0.09%); - not detected (< 0.05%). ^a^: The identified constituents are listed in their order of elution. ^b^: RI indicates the retention indices calculated against C_8_-C_40_ *n*-alkanes on the HP-5MS column. ^c^: RA indicates relative amount (peak area relative to the total peak area).

### 2.2. Antimicrobial activity

The antimicrobial activities of the essential oils of *N. chinensis* and *V. officinalis* were evaluated against 10 test microorganisms, including three Gram-positive and five Gram-negative bacteria and two fungi. Their potency was assessed quantitatively by MIC and IC_50_ values which were shown in Table 2. The two essential oils had broad spectrum antimicrobial activity. The MIC values of *N. chinensis* oil on bacteria ranged from 62.5 μg/mL to 400 μg/mL, and IC_50_ values from 36.93 μg/mL to 263.16 μg/mL. Among them, both *A. tumefaciens* and *X. vesicatoria* were the most sensitive bacteria to *N. chinensis* oil, with IC_50_ values of 36.93 μg/mL and 54.25 μg/mL, respectively. The MIC values of *V. officinalis* oil on bacteria ranged from 62.5 μg/mL to 200 μg/mL, and IC_50_ values from 40.00 μg/mL to 144.11 μg/mL. Among them, *A. tumefaciens*, *S. haemolyticus* and *B. subtilis* were the most sensitive bacteria to *V. officinalis* oil with IC_50_ values of 40.00 μg/mL, 47.37 μg/mL and 48.74 μg/mL, respectively. Antibacterial activity of the two oils was still weaker than that of the positive control streptomycin sulfate. The two oils also showed moderate antifungal activity on *C. albicans* growth and *M. oryzae* spore germination with IC_50_ values of 165.74 μg/mL and 142.59 μg/mL, respectively. As *C. albicans* was an amphotericin-resistant strain, this indicated that these two oils should be alternative ones against amphotericin-resistant *Candida* with their potential applications.

**Table 2:** Antimicrobial activity of the essential oils from *N. chinensis* and *V. officinalis*.

Test microorganism	*N. chinensis* oil		*V. officinalis* oil		Positive control^ a^	
	MIC (μg/mL)	IC_50_ (μg/mL)^ b^	MIC (μg/mL)	IC_50_ (μg/mL)^ b^	MIC (μg/mL)	IC_50_ (μg/mL)^ b^
*A* *. tumefaciens*	62.5	36.93 ± 0.51	62.5	40.00 ± 0.53	15	8.34 ± 0.09
*E* *. coli*	150	100.23 ± 0.76	200	131.88 ± 3.20	20	10.47 ± 0.31
*P* *. lachrymans*	300	236.06 ± 1.62	100	60.05 ± 0.38	15	9.01 ± 0.09
*S* *. typhi* *murium*	400	263.16 ± 2.25	200	144.11 ± 2.70	120	91.46 ± 0.55
*X* *. vesicatoria*	100	54.25 ± 0.83	125	78.16 ± 1.09	20	11.62 ± 0.19
*B* *. subtilis*	125	91.95 ± 0.09	62.5	48.74 ± 0.65	10	4.98 ± 0.06
*S* *. aureus*	200	110.62 ± 2.19	200	123.39 ± 0.66	100	78.60 ± 0.61
*S* *. haemolyticus*	100	67.13 ± 0.69	62.5	47.37 ± 0.82	15	7.75 ± 0.16
*C. albicans*	400	374.72 ± 2.46	200	165.74 ± 1.18	900	713.13 ± 1.49
*M. oryzae*	500	296.51 ± 2.75	200	142.59 ± 0.77	100	38.44 ± 0.56

^a^: The positive controls for bacteria, *Candida albicans* and *Magnaporthe oryzae* were streptomycin sulfate, amphotericin B and carbendazim, respectively. ^b^: Mean ± standard deviation of three independent experiments (six replicates for each treatment).

### 2.3. Antioxidant activity

The essential oils were subjected to screening for possible antioxidant activity by three complementary tests, namely the DPPH free radical scavenging, β-carotene/linoleic acid and ferrozine-Fe^2+^ complex formation systems [34]. The antioxidant activity results of the two essential oils are shown in Table 3. DPPH is a stable free radical which can readily experience reduction in the presence of an antioxidant. The ability of the oils to scavenge DPPH radical was determined on the basis of their concentrations with IC_50_ values of 637.47 μg/mL for *N. chinensis* oil and 493.40 μg/mL for *V. officinalis* oil, respectively.

The β-carotene bleaching assay is based on the loss of the yellow coloration of β-carotene due to its reaction with radicals formed by linoleic acid oxidation in an emulsion. The rate of β-carotene bleaching is slowed down by presence of antioxidants [36]. The two essential oils inhibited β-carotene bleaching in a dose dependent manner, with the IC_50_ values of 240.56 μg/mL for *N. chinensis* oil and 181.18 μg/mL for *V. officinalis* oil, respectively (Table 3).

Chelating activity of the essential oils was determined by the ferrozine assay [37]. Ferrozine quantitatively forms complexes with Fe^2+^. In the presence of other chelating agents, the complex formation is disrupted with the result that the red color of the complex was decreased. Measurement of the rate of red color reduction therefore allowed estimation of the chelating activity of the coexisting chelator [37]. The two essential oils inhibited the formation of ferrozine-Fe^2+^ complex in a dose dependent manner with the IC_50_ values of 231.89 μg/mL for *N. chinensis* oil and 235.44 μg/mL for *V. officinalis* oil, respectively. As shown in Table 3, antioxidant activity of *V. officinalis* oil was stronger than that of *N. chinensis* oil using either the DPPH scavenging or β-carotene bleaching assays. However, there are no obvious differences in the antioxidant activity between the two oils using the ferrozine-Fe^2+^ complex formation assay. The antioxidant activity of the oils can be better evaluated by using complementary tests with different mechanisms. Antioxidant activity of the two oils was still weaker than that of the positive controls.

**Table 3:** Antioxidant activity of the essential oils from *N. chinensis* and *V. officinalis*.

Sample	DPPH inhibition IC_50_^b^ (μg/mL)	β-Carotene bleaching IC_50_^b^ (μg/mL)	Ferrozine-Fe^2+^ complex formation IC_50_^b^ (μg/mL)
*N. chinensis* oil	637.47 ± 4.89	240.56 ± 0.66	231.89 ± 2.66
*V. officinalis* oil	493.40 ± 4.93	181.18 ± 2.82	235.44 ± 5.18
Positive control ^a^	25.66 ± 0.42	31.46 ± 0.68	18.46 ± 0.08

^a^: The positive controls for DPPH inhibition, β-carotene-linoleic acid and ferrous ions assays were BHT, BHT and EDTA, respectively. ^b^: Mean ± standard deviation of three independent experiments (six replicates for each treatment).

## 3. Experimental

### 3.1. Plant materials

Roots and rhizomes of *Nardostachys chinensis* Batal. were collected in the north of Sichuan Province of China in February 2009. The roots and rhizomes of *Valeriana officinalis* L. were collected in the centre of Henan Province of China in July 2009. They were dried in the shade at room temperature. The taxonomical identification of the plant materials was done by Dr. Zhilong Liu of China Agricultural University, where the voucher specimens (BSMPMI-200902001 and BSMPMI-200902002) of the plants were deposited.

### 3.2. Solvents and chemicals

β-Carotene, carbendazim, streptomycin sulfate, 1,1-diphenyl-2-picrylhydrazyl (DPPH) and C_8_-C_40_ *n*-alkanes were purchased from Sigma-Aldrich (USA). Linoleic acid and ferrozine disodium salt were obtained from Johnson Matthey (UK). Amphotericin B and 3-(4,5-dimethylthiazol-2-yl)-2,5-diphenyl tetrazolium bromide (MTT) were purchased from Amresco (USA). Butylated hydroxytoluene (BHT), ferrous chloride (FeCl_2_), Tween-40, ethylene diamine tetraacetic acid (EDTA) were bought from Beijing Chemical Company. All other unlabelled chemicals and reagents were of analytical grade.

### 3.3. Preparation of the essential oils

The dry roots and rhizomes of either *N. chinensis* or *V. officinalis* (1 kg) were submitted to hydro-distillation in a Clevenger-type apparatus at 100 ºC for 4 h. The distilled oil was extracted with diethyl ether and dried over anhydrous sodium sulfate. After filtration, the yields of the essential oils were 9.0 g (0.9%, w/w) for *N. chinensis*, and 7.0 g (0.7%, w/w) for *V. officinalis*. The oils were then kept in a sealed dark glass vial at 4 ºC until required.

### 3.4. Oil analysis

The composition of the essential oils of *N. chinensis* and *V. officinalis* was determined by the use of analytical GC and GC/MS techniques. The same column and analysis conditions were used for both GC and GC/MS. An Agilent 6890N Network GC system for gas chromatography was equipped with an HP-5MS column [30 m × 0.25 mm (5%-phenyl)-methylpolysiloxane capillary column, film thickness 0.25 µm], a split-splitless injector heated at 250 ºC and a flame ionization detector (FID) at 240 ºC. The oven temperature was programmed as follows: initial temperature 50 ºC for 1.50 min, increase 10 ºC/min up to 180 ºC, 2 min at 180 ºC, and then increase by 6 ºC/min up to 280 ºC, 10 min at 280 ºC. Helium (99.999 %) was used as carrier gas at a flow rate of 1.0 mL/min. The injection volume was 1.0 μL (split ratio 1:20). GC/MS analyses were performed using an Agilent 6890N Network GC system with an Agilent 5973 Network mass selective detector, mass spectrometer in EI mode at 70 eV in m/e range 10–550 amu.

The components were identified by comparison of their mass spectra with NIST 2002 library data of the GC-MS system, as well as by comparison of their retention indices (RI) with the relevant literature data [12,13,14,15,16,17,18,19,20,21,22,23,24,25,26,27]. The relative amount (RA) of each individual component of the essential oil was expressed as the percentage of the peak area relative to the total peak area. RI value of each component was determined relative to the retention times (RT) of a series of C_8_–C_40_ *n*-alkanes with linear interpolation on the HP-5MS column [28].

### 3.5. Antimicrobial activity

#### 3.5.1. Antibacterial activity assay

Three Gram-positive (*Bacillus subtilis* ATCC 11562, *Staphylococcus aureus* ATCC 6538 and *Staphylococcus haemolyticus* ATCC 29970), and five Gram-negative (*Agrobacterium tumefaciens* ATCC 11158, *Escherichia coli* ATCC 29425, *Pseudomonas lachrymans* ATCC 11921, *Salmonella typhimurium* ATCC 14028 and *Xanthomonas vesicatoria* ATCC 11633) bacteria were selected for antibacterial activity assay. They were grown in liquid LB medium (yeast extract 5 g/L, peptone 10 g/L, NaCl 5 g/L, pH 7.0) overnight at 28 ºC, and the diluted bacterial suspension (10^6^ cfu/mL) was ready for detection. A modified broth dilution-colorimetric assay by using the chromogenic reagent 3-(4,5-dimethylthiazol-2-yl)-2,5-diphenyl tetrazolium bromide (MTT) was used to detect the antibacterial activity of the essential oil [29]. Briefly, the oil was dissolved in acetone at an initial concentration of 5.0 mg/mL. Then it was diluted with 30% acetone to obtain concentrations ranging from 0.2 mg/mL to 4.0 mg/mL. Test sample solutions (10 μL) and prepared bacterial suspension (90 μL) containing 1 × 10^6^ cfu/mL were added into each well of the 96-well microplate. The negative control well contained 90 μL of the inoculum (1 × 10^6^ cfu/mL) and 10 μL of 30% acetone. Streptomycin sulfate was used as the positive control. After the plates were agitated to mix the contents of the wells using a plate shaker and incubated in the dark at 28 ºC for 24 h, 10 μL of MTT (5 mg/mL in 0.2 mol/L, pH 7.2 phosphate-buffered saline) was added into each well, and the plates were incubated for another 4 h. The minimum inhibitory concentration (MIC) value was defined as the lowest sample concentration that inhibited visible growth, as indicated by the MTT staining. Only living microorganisms could convert MTT to formazan and a blue color appeared in the well [30].

To further determine the IC_50_ value of the oil, the above microplates incubated with MTT were centrifuged at 1500 *g* for another 20 min. Then the supernatant was aspirated, 200 μL of dimethyl sulfoxide (DMSO) was added into each well, and the colored formazan products were extracted for 30 min. After complete extraction, the plate was centrifuged at 1500 g for another 20 min, and then 100 μL of the supernatant (DMSO solution) in each well was transferred to a corresponding well of another 96-well microplate to measure their light absorption values at wavelength 510 nm using the microplate spectrophotometer (PowerWave HT, BioTek Instruments, USA). The percentage (%) of the bacterial growth inhibition was determined as [(*A*_c_–*A*_t_)/*A*_c_] ×100, where *A*_c_ was an average of six replicates of light absorption values at wavelength 510 nm of the negative controls, and *A*_t_ was an average of six replicates of light absorption values at wavelength 510 nm of the samples. The median inhibitory concentration (IC_50_) was calculated using the linear relation between the inhibitory probability and concentration logarithm according to methods outlined by Sakuma [31]. The IC_50_ value was expressed as the mean ± standard deviation of three independent experiments (six replicates for each treatment).

#### 3.5.2. Antifungal activity assay

A dilution-colorimetric assay was employed to investigate the anticandidal activity of the essential oils. *Candida albicans* ATCC 10321, which was an amphotericin-resistant strain, was grown in liquid potato dextrose (PD) medium overnight at 28 ºC, and the diluted *C. albicans* suspension (10^6^ cfu/mL) was ready for assay. The final concentrations of the essential oils ranged from 0.1 to 0.5 mg/mL containing 3% (v/v) acetone. Other procedures are the same as those for antibacterial activity assay described above only amphotericin B used as the positive control and PD medium instead of LB medium.

Rice blast fungus, *Magnaporthe oryzae* (strain P131), was kindly provided by Prof. Youliang Peng of the Department of Plant Pathology, China Agricultural University. It was maintained on oatmeal-tomato agar (oatmeal 30 g/L, tomato juice 150 mL/L, and agar 20 g/L) at 25 ºC. The spores were prepared from 7-day-old cultures of *M. oryzae*, according to our previous report [32,33]. The oil-acetone solution (25 μL) was mixed with an equivalent volume of spore suspension containing 2 × 10^6^ spores per mL. The mixture was then placed on separate concave glass slides. The final concentrations of the essential oils ranged from 0.05 to 0.5 mg/mL containing 5 % (v/v) acetone. The negative control was 5% acetone, and the positive control was carbendazim at concentrations ranging from 0.05 to 0.15 mg/mL. Three replicates were used for each treatment. Slides containing the spores were incubated in a moist chamber at 25 ºC for 7 h. Each slide was then observed under the microscope for spore germination status. About 100 spores per replicate were observed to detect spore germination. The percentage (%) of spore germination inhibition was determined as [(*G*_c_–*G*_t_)/*G*_c_] ×100, where *G*_c_ is an average of three replicates of germinated spore numbers in the negative control, and *G*_t_ is an average of three replicates of germinated numbers in the treated sets. The IC_50_ value calculation for the spore germination inhibition was the same as that for antibacterial activity assay.

### 3.6. Antioxidant activity

#### 3.6.1. DPPH radical scavenging assay

Radical scavenging assay was determined by a microplate spectrophotometric method based on the reduction of a methanol solution of DPPH using the method of Ono *et al*. [34]. Briefly, DPPH solution (80 μL, 0.2 mg/mL) and essential oil solution in 30% acetone (20 μL) were added into each well of the microplate and mixed. The mixture was shaken vigorously and left to stand at 37 ºC for 30 min in the dark. The absorbance of the solution was then measured at wavelength 515 nm using a microplate spectrophotometer. Inhibition (%) of free radical (DPPH) in percent was determined as [(*A*_control_−*A*_sample_)/*A*_control_] × 100, where *A*_control_ is the absorbance of the control reaction containing all reagents except the test sample, and *A*_sample_ is the absorbance of the test oil. Tests were carried out in triplicate. BHT was used as the positive control. The IC_50_ was calculated using linear relation between the essential oil concentration and probability of the percentage of DPPH inhibition.

#### 3.6.2. β-Carotene-linoleic acid bleaching assay

The antioxidant activity of the essential oil was evaluated by β-carotene-linoleic acid bleaching method described by Ebrahimabadi *et al*. [35] with slight modifications. Briefly, linoleic acid (25 μL) and Tween-40 (200 mg) were added in the β-carotene solution (0.5 mg of β-carotene dissolved in 1 mL of chloroform). Chloroform was then removed using a rotary evaporator at 50 ºC. Fifty mL of distilled water saturated with oxygen for 30 min at a flow rate of 100 mL/min were added and the mixture was vigorously shaken. The above β-carotene-linoleic acid-Tween mixture (90 μL) and the essential oil solution (10 μL, concentrations from 1.5 mg/mL to 3.0 mg/mL) in 30% acetone solution were added into each well. An equal amount of 30% acetone was used as the control. The microplates were then placed in an incubator at 50 ºC for 2 h together with BHT as the positive control. The absorbance of the solution was then measured at wavelength 460 nm using a microplate spectrophotometer. The percentage (%) of β-carotene bleaching inhibition of each sample was determined as (*A*_β-carotene after 2 h assay_/*A*_initial β-carotene_) × 100, Where *A*_β-carotene after 2 h assay_ is the absorbance of the sample with β-carotene-linoleic acid mixture after 2 h period of incubation, and *A*_initial β-carotene_ is the absorbance of the initial mixture. All tests were carried out in triplicate. The IC_50_ value calculation for β-carotene bleaching inhibition was the same as that for antibacterial activity assay.

#### 3.6.3. Metal chelating activity on ferrous ions (Fe^2+^)

Metal chelating activity was determined according to the method of Oke *et al*. [36] with some modifications. Briefly, the essential oil in 30% acetone (20 μL) was mixed with 0.65 mmol/L FeCl_2_ and 60 μL of 4 mmol/L ferrozine disodium salt (20 μL each) in each well. Then, the mixture was shaken vigorously and left standing at room temperature for 10 min. After the mixture reached equilibrium, the absorbance of the solution was then measured at wavelength 560 nm using a microplate spectrophotometer. The ferrous ions chelating effect was calculated as the percentage (%) of inhibition of ferrozine-Fe^2+^ complex formation determined as [(*A*_control_-*A*_sample_)/*A*_control_] × 100, Where *A*_control_ is the absorbance of the only ferrozin-Fe^2+^ complex, and *A*_sample_ is the absorbance of the test essential oil and ferrozin-Fe^2+^ mixture. EDTA was used for the positive control. The IC_50_ value calculation for ferrozine-Fe^2+^ complex formation was the same as that for antibacterial activity assay.

## 4. Conclusions

We have reported the chemical composition of the essential oils of *N. chinensis* and *V. officinalis* from China along with their antimicrobial and antioxidant activities. This study revealed that both essential oils had broad spectrum antimicrobial activity on test microorganisms including bacteria and fungi. It may be partly due to the fact that they had abundant monoterpenoids and sesquiterpenoids which contributed to their antimicrobial activity and should be further studied. Antimicrobial and antioxidant properties of essential oils are of great interest in food, cosmetic and pharmaceutical industries since their possible use as natural additives emerged from the tendency to replace synthetic preservatives with natural ones [38]. The present study has provided additional data supporting the future utilization and development of *N. chinensis* and *V. officinalis* essential oils as antimicrobial and antioxidant agents. The underlying antimicrobial and antioxidant mechanisms of the essential oils as well as their active components need to be further studied and clarified.
